# An Early Instance of Upper Palaeolithic Personal Ornamentation from China: The Freshwater Shell Bead from Shuidonggou 2

**DOI:** 10.1371/journal.pone.0155847

**Published:** 2016-05-26

**Authors:** Yi Wei, Francesco d’Errico, Marian Vanhaeren, Feng Li, Xing Gao

**Affiliations:** 1 Key Laboratory of Vertebrate Evolution and Human Origins of Chinese Academy of Sciences, Institute of Vertebrate Paleontology and Paleoanthropology, Chinese Academy of Sciences, Beijing, China; 2 Department of Earth Science, University of Chinese Academy of Sciences, Beijing, China; 3 Centre National de la Recherche Scientifique, UMR 5199 - PACEA, Université de Bordeaux, Pessac, France; 4 Evolutionary Studies Institute and DST-NRF Centre of Excellence in Palaeosciences, School of Geosciences, University of the Witwatersrand, Johannesburg, South Africa; Universidade do Algarve, PORTUGAL

## Abstract

We report the discovery and present a detailed analysis of a freshwater bivalve from Shuidonggou Locality 2, layer CL3. This layer is located c. 40 cm below layer CL2, which has yielded numerous ostrich eggshell beads. The shell is identified as the valve of a *Corbicula fluminea*. Data on the occurrence of this species in the Shuidonggou region during Marine Isotope Stage 3 and taphonomic analysis, conducted in the framework of this study, of a modern biocoenosis and thanatocoenosis suggest that the archeological specimen was collected at one of the numerous fossil or sub-fossil outcrops where valves of this species were available at the time of occupation of level CL3. Experimental grinding and microscopic analysis of modern shells of the same species indicate that the Shuidonggou shell was most probably ground on coarse sandstone to open a hole on its umbo, attach a thread, and use the valve as a personal ornament. Experimental engraving of freshwater shells and microscopic analysis identify an incision crossing the archaeological valve outer surface as possible deliberate engraving. Reappraisal of the site chronology in the light of available radiocarbon evidence suggests an age of at least 34–33 cal kyr BP for layer CL3. Such estimate makes the *C*. *fluminea* recovered from CL3 one of the earliest instances of personal ornamentation and the earliest example of a shell bead from China.

## Introduction

Personal ornaments are generally considered as one of the most reliable proxies for the emergence of symbolic material culture in our genus (e.g. [1–6]). They represent in all current and historically known human societies a quintessential means of communication to convey coded information on the social and group identity of the wearer [7–13]. They may also signal the emergence of uniquely human cognition and complex language abilities [14–16] although this view remains controversial [17–20]. Although the tempo and mode of the emergence and diversification of personal ornaments is key for understanding changes in our ancestors’ cognition and behavior, such a process remains poorly known in many regions of the world.

Here we report the discovery, and present a detailed analysis of a perforated and possibly incised freshwater bivalve from Shuidonggou Locality 2, China, and discuss its significance in the framework of what is known on the earliest evidence of bead use in Africa, Europe, Asia and Australia. The object comes from a layer underlying an ostrich eggshell bead (OESB) rich horizon dated to 31–30 cal kyr BP [21–23], which makes this shell one of the earliest examples of personal ornaments from China. The purpose of this paper is to document evidence of natural, anthropogenic and post-depositional modifications present on this object, and reconstruct, based on reference collections and experimental data created for this study, the processes involved in its acquisition, modification and use. In addition, we wish to discuss the age of the cultural layer that has yielded the object, and explore the implications of this and previous discoveries for the identifications of cultural trends in the Chinese Late Palaeolithic.

### The earliest evidence for bead use in Africa, Europe, Asia and Oceania

The earliest known examples of personal ornaments are found in North Africa and Sub-Saharan Africa (Table 1 and references therein). They consist of perforated marine gastropods and bivalves [24–25]. The small gastropods *Nassarius gibbosulus* and *N*. *circumcinctus* were used as beads at seven Aterian sites from Morocco and Algeria dated to 135–60 ka. These species and *Glycymeris* sp. bivalves are identified as beads at two Western Asian, Levantine Mousterian, sites dated to c. 100 ka.

**Table 1:** Earliest known instances of shell beads.

Site	Country	Age (ka)	Species	Number	Cultural attribution	Reference
Qafzeh	Israel	80–100	*Glycymeris insubrica*	10	Levantine Mousterian	[24][25]
Skhul	Israel	100–135	*Nassarius gibbosulus*	2	Levantine Mousterian	[30]
Oued Djebbana	Algeria	90	*Nassarius gibbosulus*	1	Aterian	[30]
Taforalt	Morocco	82	*Nassarius gibbosulus*	29	Aterian	[31][32]
			*Nassarius circumcinctus*	3	Aterian	
Rhafas	Morocco	70–80	*Nassarius gibbosulus*	3	Aterian	[32]
			*Nassarius circumcinctus*	1	Aterian	
			*Columbella rustica*	1	Aterian	
Ifri n’Ammar	Morocco	82	*Nassarius gibbosulus*	1	Aterian	[32]
			*Columbella rustica*	1	Aterian	
Contrebandiers	Morocco	96–107	*Nassarius gibbosulus*	many	Aterian	[32][33][34]
Contrebandiers	Morocco	115–122	*Nassarius gibbosulus*	many	Mousterian	[32][33][34]
Blombos	South Africa	75	*Nassarius kraussianus*	41	Still Bay	[35][36][26]
Sibudu	South Africa	70	*Afrolittorina africana*	3	Still Bay	[37]
Border Cave	South Africa	74	*Conus ebraeus*	2	Howieson’s Poort	[38][39]

Perforated *Nassarius kraussianus*, *Conus ebraeus* and *Afrolittorina africana* shells are found at Still Bay and Howiesons Poort sites from southern African dated to c. 75 ka. Many of these shells occur at sites located far from the coast, bear traces of pigment, intense use-wear indicative of a prolonged utilization, and appear to have been blackened by heating, probably to change their natural color [26]. Personal ornaments, consisting of circular ostrich egg shell and, more rarely, stone beads reappear in Africa at c. 50 ka [2, 27–29].

In Europe, the oldest evidence of a possible use of shells as beads comes from two Mousterian sites dated to ca. 50 ka. *Acanthocardia* sp. and two valves of of Glycymeris sp., with natural perforations and a valve of Spondylus sp., with traces of red pigment were found at Cueva de Los Aviones [40]. At Fumane, in Italy, a fossil gastropod with traces of use-wear and hematite rich pigment is interpreted as a broken bead or a manuport [41]. At seven other Mousterian sites dated to between 130 ka and 44 ka, large raptors phalanges bearing cut-marks are interpreted as personal ornaments, and cut-marks on wing bone as evidence for the extraction of large feathers for symbolic purposes [42–47].

After 45 ka, and particularly after 42 ka, personal ornaments are found at numerous sites from Europe and Western Asia. At most sites of this period from Europe, ornaments differ from their antecedents in that they are manufactured from a broad variety of raw materials (teeth, bone, ivory, stone, many living and fossil shell species etc.) and often take the form of dozens of discrete types [13, 48–50].

Although evidence for the use of personal ornaments in the time range of Early Upper Palaeolithic of Europe is still reduced in Asia and Oceania, it shows a contrasted pattern [51]. The earliest known ornaments from Oceania reveal a focus on marine resources. The oldest personal ornament from this large region is the perforated tiger shark tooth from Bang Merabak, New Ireland, recovered in a layer dated to between 39.5–28 ka BP [52]. The earliest evidence for bead use in Australia comes from the site of Mandu Mandu, Cape Range of Western Australia, where 22 *Conus* sp. shell beads were recovered in a layer dated to ca. 32 ka [53]. In addition, 10 Dentaliidae shell beads are reported from the 30 ka old layers of Riwi in the Kimberley of Western Australia, a site located 300 km inland [54].

In contrast, Early Upper Palaeolithic (EUP) sites from Asia yield personal ornaments that associate objects comparable in raw material and type diversity to their contemporary counterparts from Europe and Western Asia (perforated mammal teeth, beads and pendants made of ivory, bone, stone) with types specific to this large region: OESB, bone tubes decorated with circular notches, and perforated freshwater shells. At Yafteh, Iran, ornaments found in layers dated to 38.4–37.8 cal kyr BP consist of perforated deer canines, imitation of these teeth in hematite, and marine shells [55]. In Siberia and Mongolia OESB are found in initial Upper Palaolithic layers at Denisova, Tolbor 4/AH 6–5, Tolbor 16, Dörölj 1/13, Khotyk/A-H 2 (40–28 ^14^C ka BP), Khotyk/A-H 3 (34–26 ^14^C ka BP), and Podzvonkaya; decorated bone tubes bearing parallel incisions at Kamenka A/3 and Denisova in layers ranging from 40 to 35 cal kyr BP; bone pendants dated to 43.3 ± 1.6 ^14^C BP at Kara Bom; perforated freshwater shells at Denisova and Anui 2 [56–69]. OESB are also found at Patne, in India, in layers dated to 30 cal kyr BP [70]. Shell beads have been found at Batadomba-lena Cave, Sri Lanka, in layers dated to 35–30 cal kyr BP [71].

A comparable trend is observed in China. At Xiaogushan (Liaoning), a perforated canine of *Nyctereutes* sp. and another of *Felis chinensis* were found in layer 2, possibly dated to 33–43 kyr cal BP [72–73]. A perforated red deer canine, a carnivore canine, and a fragment of bone disc decorated with notches were unearthed from the lower part of layer 3 of this site [72], recently re-dated by AMS ^14^C and OSL at 30–20 ka BP [73]. At Zhoukoudian Upper Cave [74] an array of ornaments primarily consisting of perforated teeth from a variety of mammals and, to a lesser extent, bone tubes, marine shells, stone beads and perforated pebbles come from layers dated between 29 and 11 cal kyr BP [75–79]. Perforated and grooved teeth are found at Yuchanyan (Hunan) in layers dated to c. 18–13 cal kyr BP [80].

A deliberately perforated stone bead made of graphite is found at the Shiyu (Shanxi), a site dated to 33–31 cal kyr BP [81]. An elongated piece of limestone presenting a natural perforation and scars tentatively interpreted as due to suspension was recovered at Xiaonanhai, Henan Province, in layers dated to between 15 and 13 cal kyr BP [82–83]. A perforated object, reported by Qu et al. [84] as a bone pendant, by Mei [85] as a stone pendant, but described by Xie et al. [86] as a possible pendant made of bone or stone, is found at Ma’ashan (Hebei) in layers dated to 15.5–16.4 cal kyr BP [86].

Two Chinese sites significantly differ from the previous ones and those from Siberia. Only OESB were so far recovered from Shuidonggou Locality 1, 2, 7 and 8, in layers dated to c. 31.3–29.9 cal kyr BP, 24±2 to 30±3 ka (OSL), and 31.3±0.1 cal kyr BP respectively [22, 87]. At Shizitan (Shanxi Jixian), OESB also dominate the picture. They are found at five localities (S1, S9, S12G, S24, S29) in layers dated to between 25 and 11.3 cal kyr BP [88]. At Locality S29 (24.9–18.8 cal kyr BP) they are found in association with an *Anadara kagoshimensis* perforated on the umbo. At Locality S12A (19.5–18.9 cal kyr BP) OESB are associated with an unidentified bivalve fragment presenting two perforations, at Locality S9 (11.8–11.3 cal kyr BP) with two Verenidae shells perforated on the umbo, an elongated unidentified bivalve perforated close to its margin, and a bone tube. Finally, a site called Yujiagou within the Late Palaeolithic site complex of Hutouliang (Hebei), has yielded, in layers dated to c. 12–10 cal kyr BP, both types of ornaments: OESB and perforated gastropods on the one hand, bone tubes, stone pendants, and a perforated shell disc, on the other hand [85–86]. At other Hutouliang’s localities, dated to 13–12 cal kyr BP, OESB and perforated shells are associated with bone and stone beads [89–90].

### Archaeological Context of Shuidonggou Locality 2

**Fig 1:**
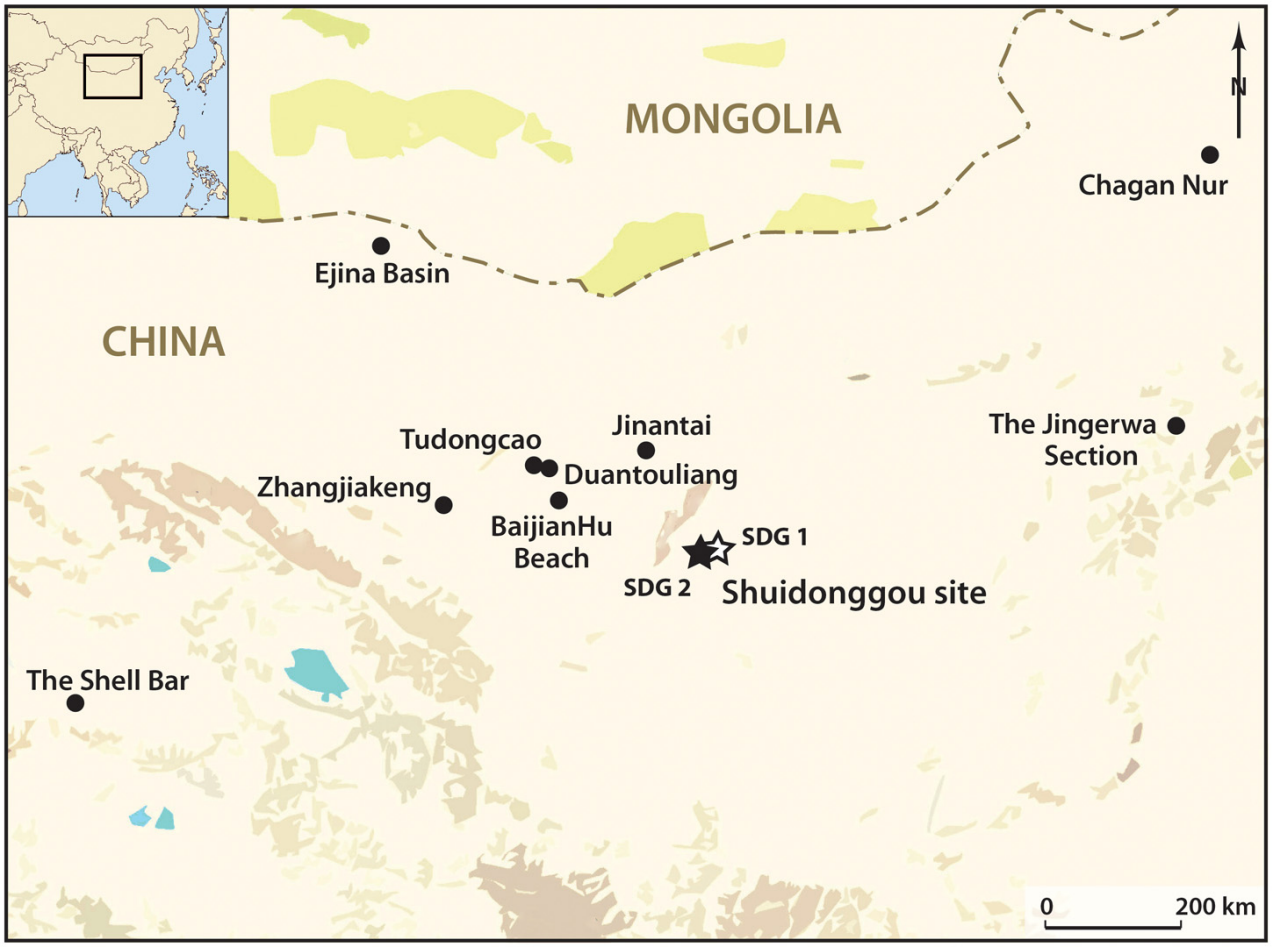
Location of the site and of lacustrine fossil deposits where *Corbicula* shells were found. Stars: Shuidonggou site complex (38°17´N, 106°30´E); 2. BaijianHu Beach (39°09´N, 104°10´E); 3. Tudongcao (39°33´N, 103°49´E); 4. Duantouliang (39°34´N, 103°57´E); 5. Zhangjiakeng (38°56´N, 102°15´E); 6. Jinantai (39°50´N, 105°35´E); 7. Ejina Basin (42°21´N, 101°15´E); 8. the Jingerwa section (40°06´N, 114°20´E); 9. Chagan Nur (43°25´N, 114°55´E); 10. The Shell Bar (36°30´N, 96°12´E).

**Fig 2:**
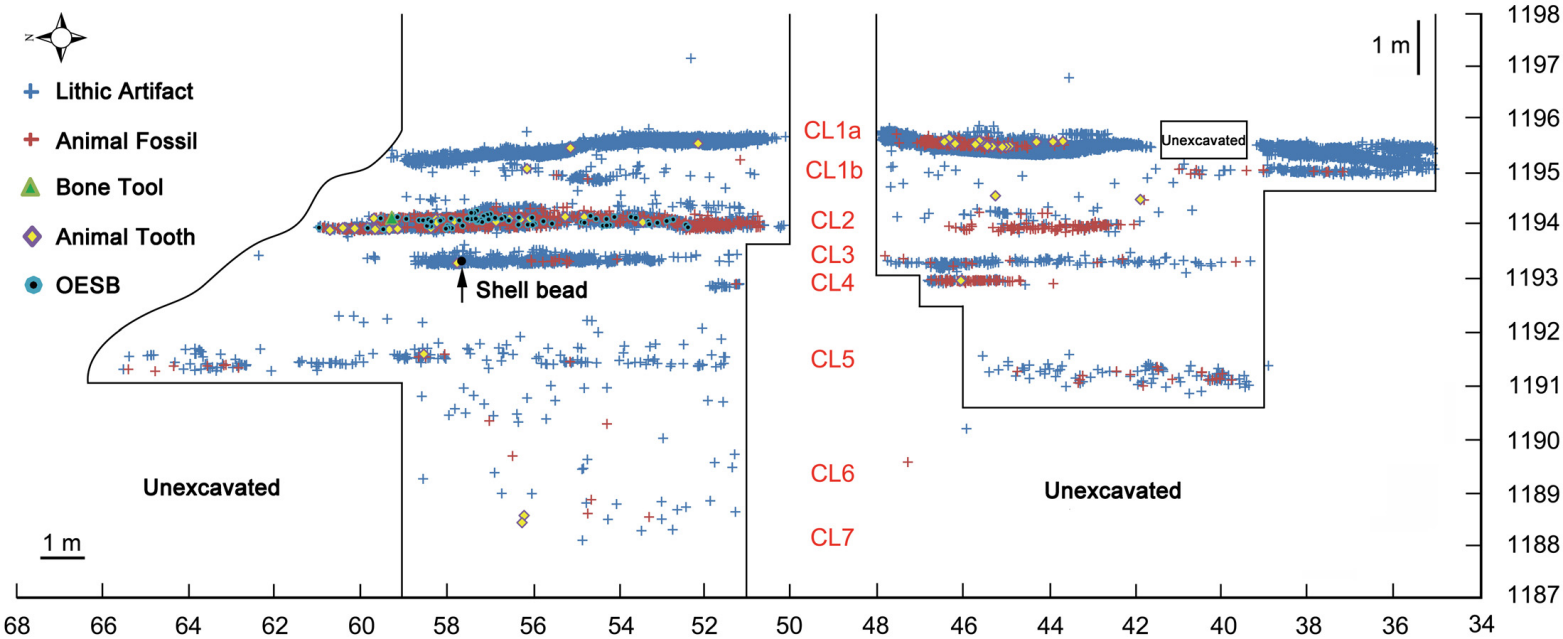
Vertical distribution of selected finds categories at Shuidonggou Locality 2. Cultural layers (CL) and the location of the shell analysed in this study are indicated. Vertical and horizontal scales in metres. Reprinted from Chen et al. [93] under a CC BY license, with permission from Acta Anthropologia Sinica, original copyright 2012.

Shuidonggou Locality 2, one of twelve sites composing the Shuidonggou archaeological site complex, is situated on the second terrace of the Huanghe River, 28 km southeast of the town of Yinchuan, in northern China (Fig 1). The area is situated on the southwestern margin of the Ordos Desert which, climatically, is at the transition between the arid desert and the semi-arid Loess Plateau. Locality 2 was first discovered in 1923 by Licent and Teilhard de Chardin [91]. Systematic excavation by a team of the Institute of Vertebrate Paleontology and Paleoanthropology, Chinese Academy of Sciences, took place in 2003–2005 and 2007 over a surface of 100 m^2^ and a depth of 12.5 m. Eighteen stratigraphic layers (Fig 2) were recognized, seven of which contain Palaeolithic archaeological assemblages in the form of rich lithic industries, faunal remains, and 83 OESB [22, 92].

The sequence was dated by ^14^C and OSL methods (Table 2). The latter, however, contributes little to clarify the chronology of the occupation due to their large standard error and almost systematic discrepancy with ^14^C determinations. Radiocarbon chronology reported by Li et al. [96] proposes that Locality 2 was occupied 41.4–34.4 cal kyr BP (layer CL7), 34.4–32.6 cal kyr BP (CL6 and 5), 32.6–31.4 cal kyr BP (CL4 and 3), 31.3–29.9 cal kyr BP (CL2), and perhaps, based on a single OSL age, 20.3±1.0 ka (CL1). This chronology has been recently discussed by Keates and Kuzmin [97] (see reply by Li et al. [98]), who use the large dispersion of ^14^C determinations to challenge the idea of a precocious appearance of a blade technology at Locality 2.

**Table 2:** Radiometric dating of Shuidonggou Locality 2 (data after Gao et al. 2013, Li et al. 2013).

Cultural layer	Provenance	Context	Material	Dating method	Lab #	14C Age BP	Cal BP, (95.4%)/OSL*	References
1	Cultural layer 1	Profile	Sediment	OSL	IEE1880		20300±1000	[92]
2	Hearth 1	Profile	Charcoal	AMS	Beta-132982	26350±190	31012–30203	[94] [95]
2	Hearth 2	Profile	Charcoal	AMS	Beta-132983	25670±140	30339–29414	[94] [95]
2	Hearth 2	Profile	Ostrich eggshell	AMS	Beta-132984	26930±120	31207–30818	[94] [95]
2	Hearth 3	Profile	Charcoal	AMS	Beta-134824	26830±200	31220–30699	[94] [95]
2	Hearth 4	Profile	Charcoal	AMS	Beta-134825	25650±160	30365–29365	[94] [95]
2	Hearth 5	Profile	Charcoal	AMS	Beta-146355	26310±170	30975–30204	[94] [95]
2	Hearth 7	Profile	Charcoal	AMS	Beta-146357	29520±230	34125–33230	[94] [95]
2	Hearth 10A	Profile	Charcoal	AMS	Beta-146358	23790±180	28283–27572	[94] [95]
2	Cultural layer 2	Profile	Ostrich eggshell	AMS	Beta-207935	28420±160	32918–31719	[21]
2	Cultural layer 2	Profile	Charcoal	AMS	Beta-207936	28330±170	32822–31620	[21]
2	2L3	In situ	Charcoal	AMS	BA110217	26450±120	30996–30492	[93]
2	L18	In situ	Charcoal	AMS	BA110218	30360±120	34656–34056	[93]
2	L20-H6	In situ	Charcoal	AMS	BA110219	25090±90	29441–28844	[93]
2	2L4	In situ	Charcoal	AMS	BA110220	26040±90	3070 7–29911	[93]
2	L20-H7	In situ	Charcoal	AMS	BA110221	2520±30		[93]
2	L21-H7	In situ	Charcoal	AMS	BA110226	895±30		[93]
3	L28	In situ	Bone	AMS	BA110222	27190±100	31324–30965	[93]
3	L27	In situ	Bone	AMS	BA110223	28290±110	32665–31655	[93]
3	Cultural layer 3	Profile	Sediment	OSL	IEE1881		27800±1400	[92]
4	Cultural layer 4	Profile	Sediment	OSL	IEE1882		20500±1100	[92]
4	L30	In situ	Charcoal	AMS	BA110224	985±30		[93]
5	Cultural layer 5	Profile	Sediment	OSL	IEE1883		29200±2100	[92]
5	Cultural layer 5	In situ	Bone	AMS	BA110227	20280±70	24569–24108	[93]
6	Upper part	Profile	Sediment	OSL	IEE1884		23600±2400	[92]
6	Low part	Profile	Sediment	OSL	IEE1885		38300±3500	[92]
7	Upper part	Profile	Peat	AMS	BA07940	29759±245	34351–33490	[92]
7	Low part	Profile	Wood	AMS	BA07943	36329±215	41475–40441	[92]
7	Peat deposit	In situ	Wood	AMS	BA110228	980±30		[93]

* 14C dates were calibrated using OxCal 4.2 online software (IntCal 13 curve).

In the framework of the present study we have conducted an updated calibration (Table 2), of the ^14^C ages from this site using OxCal 4.2.4 and the IntCal13 curve [99, 100]. Attempts to produce a Bayesian age model failed because of the chronological inversions between cultural layers.

The shell described in this study was recovered in layer CL3. The shell inventory number is SDG2:T2-9736 and its coordinates are the following: X = 50.199m, Y = 57.725m, Z = 1193.316m. No fresh water shells were found in the other cultural layers excavated at Shuidonggou Locality 2. The object was found close to stone artifacts, animal bone fragments, and teeth in a yellowish sand lens. The retouched stone tools found in the same layer are typologically and technologically characteristic of the northern Chinese Late Palaeolithic. They are usually made by simple hard hammer percussion and occasionally by bipolar reduction. The high degree of fragmentation makes the taxonomic attribution of the faunal remains from this layer difficult. Mammal teeth found in the overlying layer CL2 belong to *Equus przewalskyi*, *Equus hemionus*, and *Gazella przewalskyi*.

## Material and Methods

### Taxonomic identification

The shell described in this study is curated at the Institute of Vertebrate Palaeontology and Paleoanthropology (IVPP), Chinese Academy of Sciences, Beijing. The shell was identified using criteria proposed by Ruppert et al. [101]. Identification was confirmed by Zhang Hucai, Yunnan Normal University, a specialist of the genus *Corbicula* and its occurrence in Northwest China Quaternary sequences.

### Reference collection

**Fig 3:**
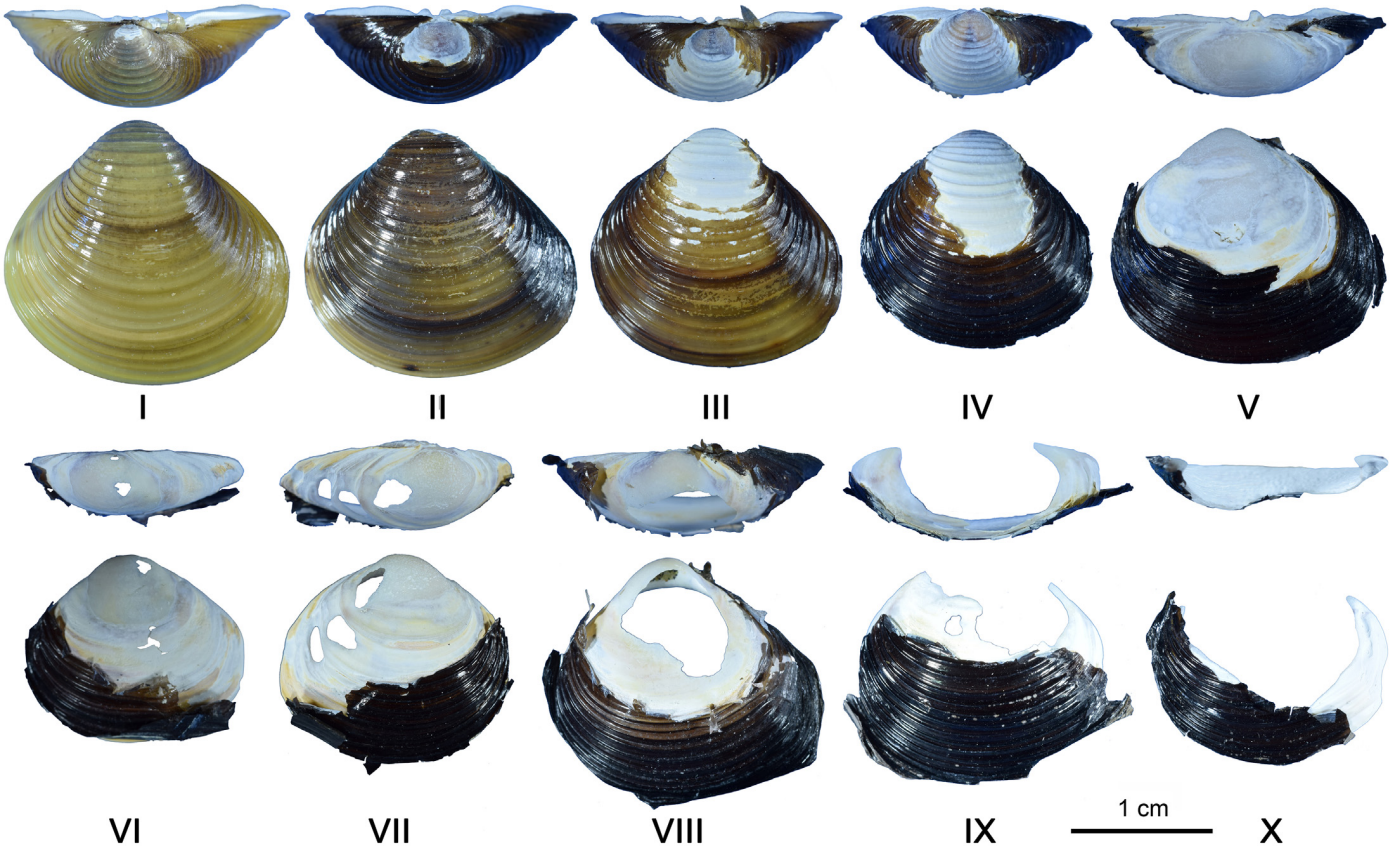
Stages of degradation recorded on the *Corbicula fluminea* reference collection created for this study.

A reference collection comprising 169 living and 1153 dead *Corbicula fluminea* was created by three people, including two of us (YW, FD) in June 2015 at the Ispe et Biscarrosse lake, France (44°26'29.2"N 1°11'18.2"W). No permit to collect Corbicula shells, which is an invasive species, is necessary according to French regulation (Article L. 411–1 du Code de l'Environnement and Arrêté Ministériel of the 23rd of April 2007). We provide a letter of the Direction Régionale de l'Environnement, de l'Aménagement et du Logement in which it is stated that no permit is required to collect this species. We also provide the text of the French law in which it clearly appears that *Corbicula* is not among the protected genera. Shells were systematically collected for three hours by surveying and snorkeling at four spots along a transect perpendicularly crossing the lake shore: on the beach, and between 0–30 cm, 30–100 cm, and 100–200 cm of water depth. We also recovered dead and living shells by sieving 1 m^2^ of superficial sand with a 0.5 mm mesh sieve at the 0–30 cm water depth spot. Living shells were put in hot water, opened and cleaned. Ten stages of degradation (Fig 3) were recorded on living and dead *Corbicula fluminea*: I, living shells; II-IV, dead shells with increasing darkening and flaking off of the periostracum; V-VIII, erosion of the ostracum and appearance of perforations in the hypostracum, IX-X; disappearance of the umbo and an increasing proportion of the shell body.

### Experimental perforation and engraving

**Fig 4:**
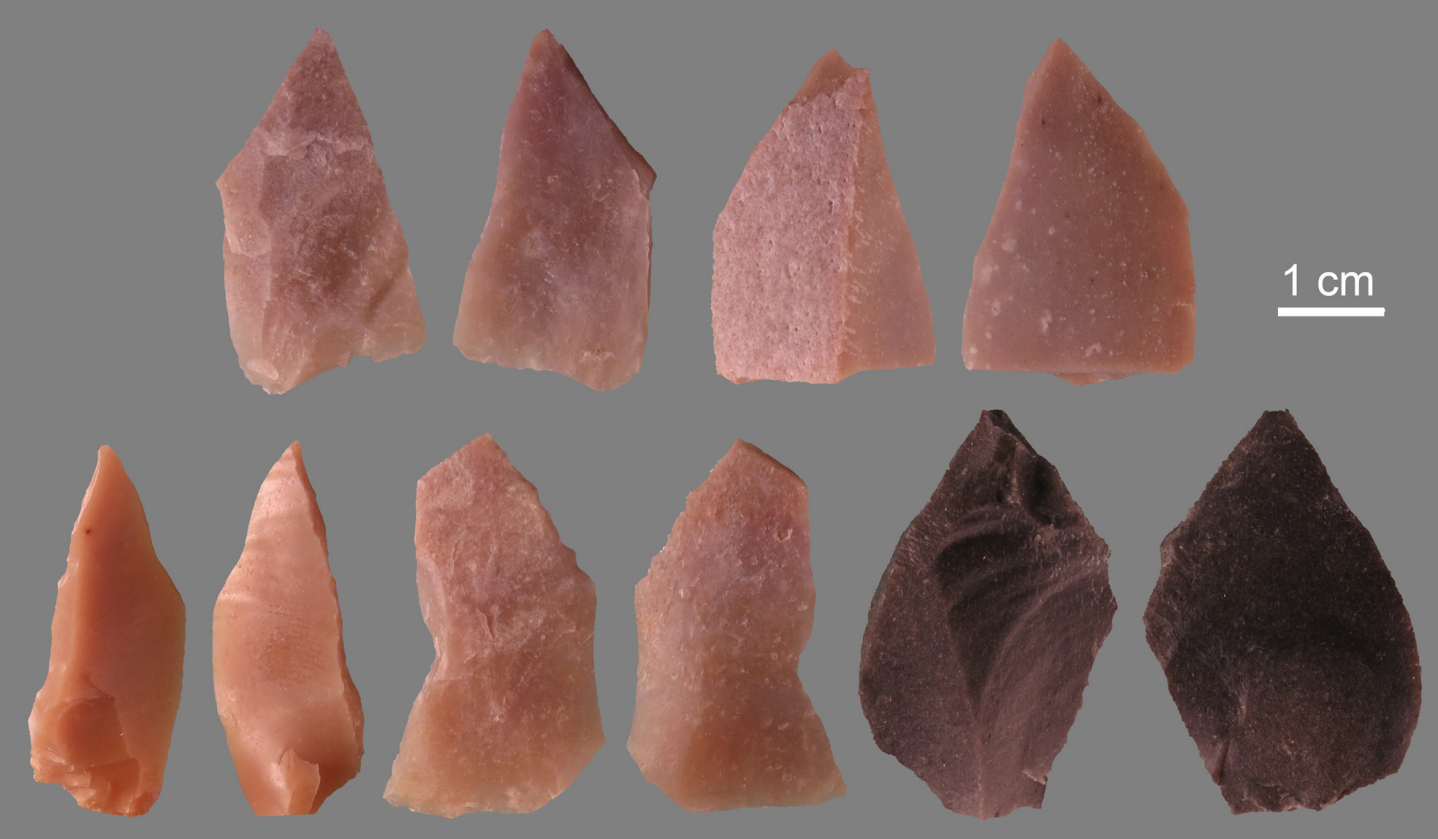
Experimental stone tools. Photo of both sides of flint tools used to experimentally engrave *Corbicula fluminea* and *Spisula solida* valves.

Umbos of well-preserved *Corbicula fluminea* were rubbed against grindstones made of basalt, granite, coarse- and fine-grained sandstone. During the experiment the shell was held between the thumb and index finger, and displaced back and forth along a direction perpendicular to the bivalve axis of symmetry until opening of a perforation. Straight lines running from the umbo to the ventral margin were engraved with flint burins (Fig 4) on well preserved valves of *Corbicula fluminea* and *Spisula solida*. The latter is a marine species with a concentric sculpture similar to that observed on the archaeological specimen and less prominent than that characteristic of well preserved *Corbicula fluminea* valves.

### Microscopic analysis

The shell was studied and photographed at the IVPP using a Wild M3C binocular equipped with a Coolpix 990 digital camera and a Zeiss MA EVO25 scanning electron microscope. Experimentally engraved and ground *Corbicula fluminea* were examined with a motorized Z6 APOA equipped with a DFC420 digital camera. Uploaded images were treated with Leica Application Suite (LAS) equipped with Multifocus module, and Leica Map DCM 3D software. The Multifocus module produces extended depth of field images. Once digital images are collected at different heights, adapted algorithms combine them into one single, sharp composite image that significantly extends the depth of field. Treatment of data by the Leica Map DCM 3D produces 3D reconstructions of areas of interest. Selected areas of experimentally ground and engraved shells were also scanned using a Sensofar Sneox scanning confocal microscope equipped with a 20x objective. Resulting data were treated with SensoSCAN 6.0 software to obtain 3D renderings of the surfaces. Tracings of anthropogenic modifications identified on the shell were made with Adobe Illustrator software on macroscopic and microscopic photographs taken under incident light.

## Results

### Taxonomic identification

**Fig 5:**
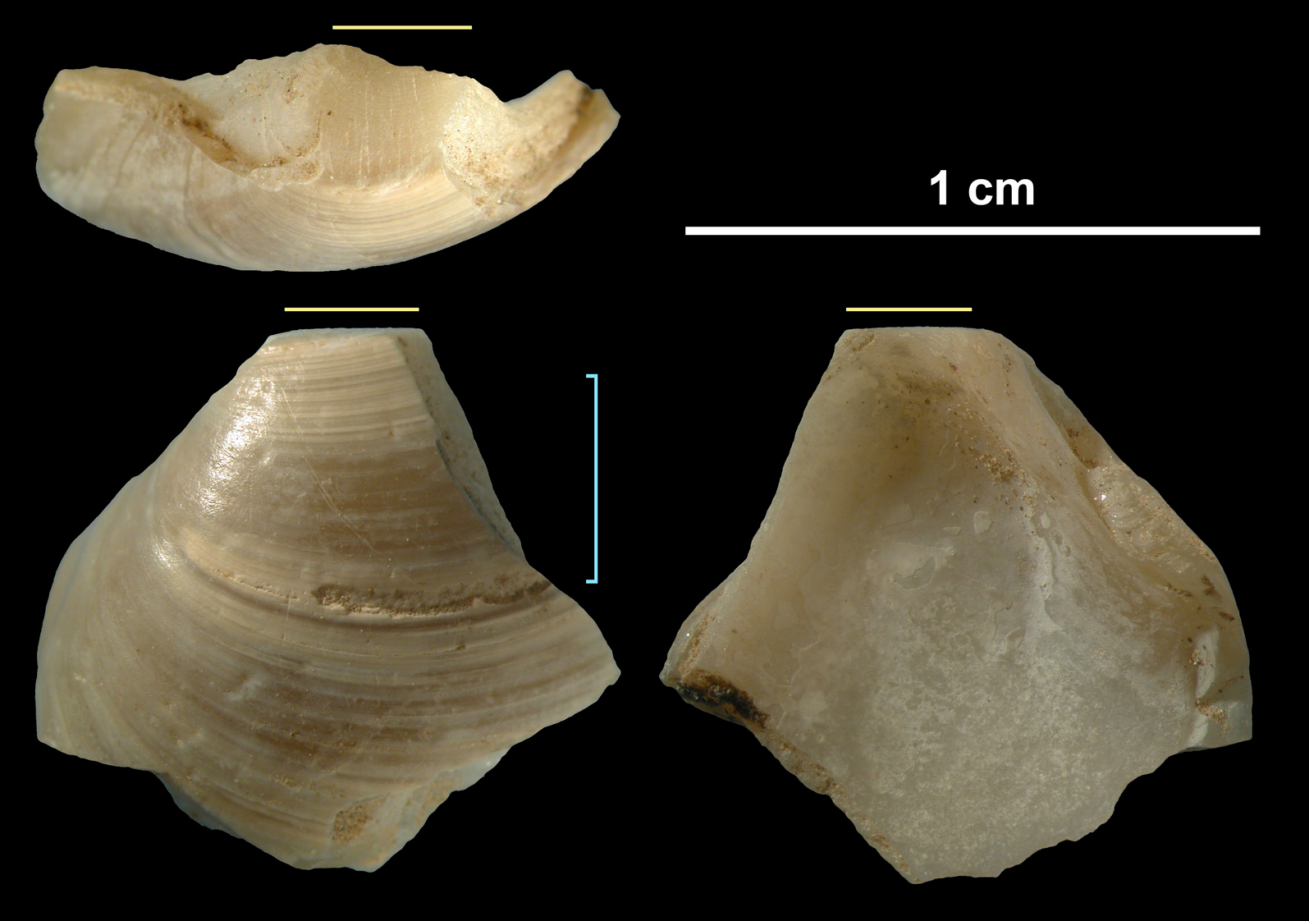
Broken shell bead from Shuidonggou Locality 2, Cultural layer 3. Horizontal yellow lines indicate the location of the ground facet. The blue vertical line identifies the area where the incised line is observed.

The object described in this study (Fig 5) is identified as the left valve of a *Corbicula fluminea* (Müller 1774) [102].

Commonly named Asian clam, this freshwater mollusk belongs to the family of the Cyrenidae [101]. Native to South and East Asia, this species is nowadays invasive in many aquatic environments where it is threatening and replacing local taxa [103–105]. Modern *C*. *fluminea* lives in areas with temperate to warm, humid climate with annual average precipitation higher than 800 mm, generally ranging from 800 to 1400 mm, and average annual temperature of 13.5–17°C [106]. However, it can survive temperatures of 2 to 36–37°C [107–108].

### Shell provenance

*C*. *fluminea* do not live in rivers and lakes close to the Shuidonggou site at present [106]. This genus, however, was certainly ubiquitous in lacustrine and fluviatile environments of the region during Marine Isotope Stage (MIS) 3. Fossil freshwater shells attributed to C. *fluminea* are reported [109] from ten paleobeach and paleolake deposits (Fig 1) located in the Tengger Desert (BaijianHu Beach, Tudongcao, Duantouliang, Zhangjiakeng), the Wulanbuhe Desert (Jinantai), the Ejina Basin along the border between China and Mongolia, the Nihewan Basin (Jingerwa), and the Qaidam Basin (Chagan Nur, Shell Bar). *Corbicula* represents a stratigraphic marker of these deposits, dated to the late MIS3 [109]. The outcrops from the Tengger Desert, ^14^C dated to 41–25 cal kyr BP [110–111], are 200 km away from Shuidonggou. This implies that the *Corbicula* from layer CL3 may have been collected alive or dead in freshwater environments close to Shuidonggou or at more distant outcrops containing fossil specimens.

### Taphonomy

Valves of *Corbicula* are composed of three layers: the periostracum, an outer organic coating consisting of conchiolin fibers that takes a yellowish to brownish color; the ostracum made of hexagonal prisms of calcite arranged perpendicularly to the shell surface; and the hypostracum, an inner pearly layer consisting of lamellae of aragonite parallel to the valve surface [112, 101].

**Fig 6:**
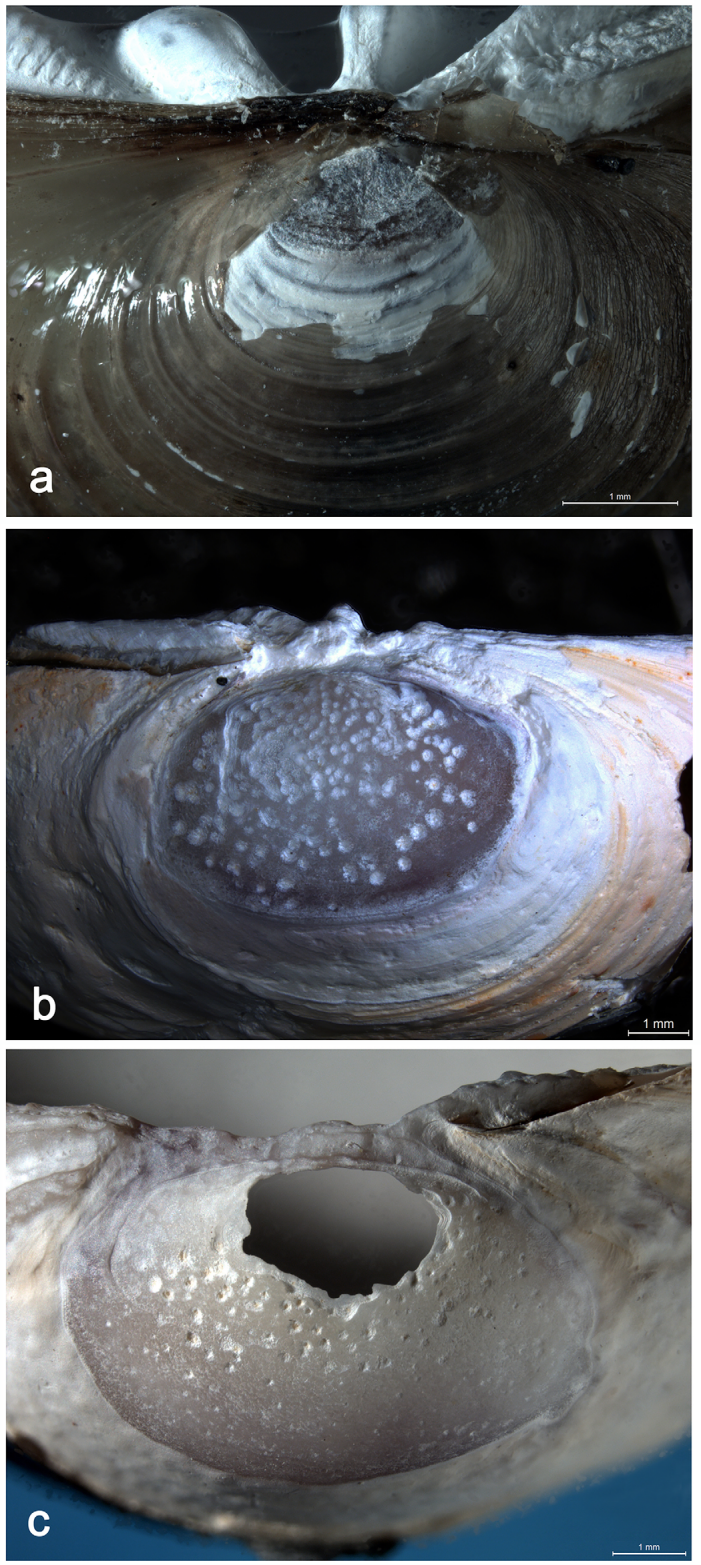
Umbos of *Corbicula fluminea* shells at different stages of degradation. **a.** Stage I, living shell; **b.** Stage IV; **c.** Stage VI.

Our survey indicates that living Asian clam shells can be easily collected at less than 1 m depth in freshwater environments colonized by this genus (Table 3). Valves of dead shells at different stages of decay are also effortlessly available in these environments, on beaches and shallow waters. Living and dead shells from these assemblages, however, markedly differ in their appearance from the archaeological specimen. Living and well preserved dead specimens (stage II-III) display projecting growth lines that appear instead highly and homogeneously smoothed on the Shuidonggou piece. In 95% of the living *Corbicula* shells collected during our survey the periostracum is missing at the tip of the umbo, which causes localized erosion of the underling ostracum, and the appearance of microscopic pits (Fig 6a).

**Table 3:** Occurrence of modern *Corbicula fluminea* at different stages of degradation.

Location/depth (cm)	Stages of degradation *										Total
	I	II	III	IV	V	VI	VII	VIII	IX	X	
beach	3	196	77	10	5	8	7	0	41	10	357
0–30	14	230	96	9	2	4	1	0	1	1	358
0–30**	7	170	82	12	4	5	0	1	1	3	285
30–100	136	28	20	13	14	16	7	6	15	7	262
100–200	9	0	4	3	19	6	5	30	5	1	82
Total	169	624	279	47	44	39	20	37	63	22	1344

** I: living specimens; II-X: progressive stages of degradation

* shells recovered from sieving the sand

Such alteration remains very superficial and never leads, at this stage, to the perforation of the umbo and the appearance of the striated facet observed on the archaeological specimen.

Valves at an initial stage of alteration (stages II-IV) associate well preserved growth lines with removal of the periostracum and the ostracum on the umbo, and the appearance of extensive pitting on the hypostracum in this area (Fig 6b). Following stages are characterized by the loss of the periostracum and ostracum on a wider area (stage V) and, subsequently, by the opening of perforations (Fig 6c) in the hypostracum (stage VI-VIII), and the eventual fracture of the valve producing typical moon-shaped fragments (IX-X). None of the valves from the sampled thanatocoenosis display the clean fractures splitting the archaeological specimen.

**Fig 7:**
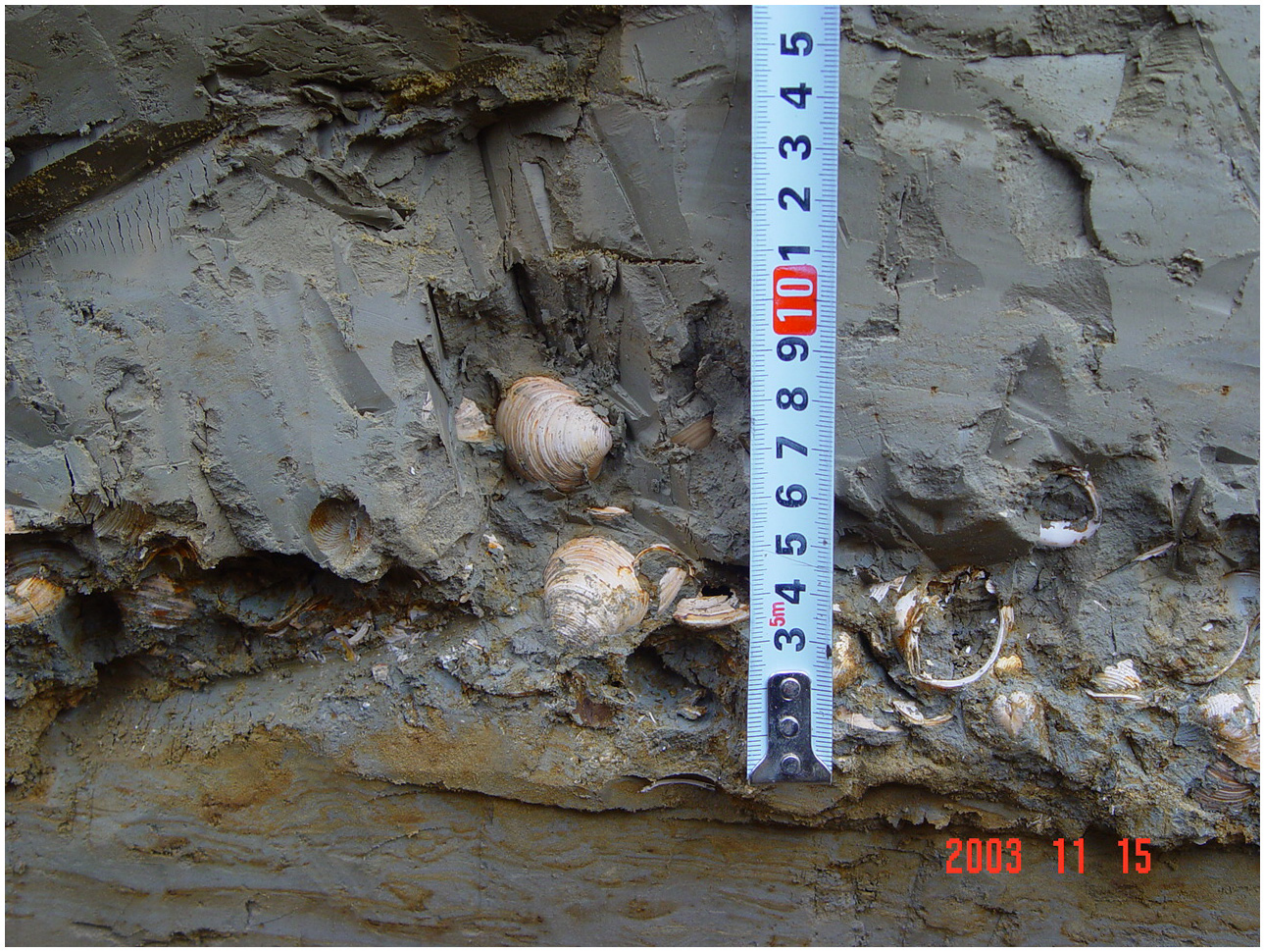
Freshwater shells visible in the Shell Bar Section, Salt Lake Qarhan, Qaidam Basin (photo H. Zhang). Reproduced with the permission of the author.

Natural perforations affecting, at different degrees, *Corbicula* valves (stages VI-VIII) only occur on dead specimens and are observed on 10% of the valves from the sampled thanatocoenosis (Table 3). These perforations significantly differ from the incomplete perforation observed on the Shuidonggou specimen. They have irregular outlines, linear thin edges linked to the thinning and weakening of the shell wall, are surrounded by large areas in which the ostracum is removed by decalcification, and generally occur on specimens in which at least one other perforation is also present on the valve. None of them displays the striated facet flattening the umbo tip seen on the archeological specimen. Likewise, no grooves comparable to those identified on the outer surface of the archaeological valve are observed on living and dead specimens of our reference collection. In summary, the Shuidonggou valve displays a number of modifications, absent in our reference collection, which are described in detail below. Its state of preservation is compatible with that of a living or freshly dead mollusk subtracted to the gradual process of alteration affecting the valves of this species in calm shallow waters. The archaeological shell appears to have been submitted instead to a mild mechanical abrasion followed by preservation in a sedimentary environment that has prevented chemical etching of the calcitic shell layers. This is consistent with the hypothesis that the shell was collected at a fossil or sub-fossil outcrop rather than in water or along beaches. Available information on the state of preservation in which *Corbicula* valves are found at fossil outcrops close to Shuidonggou supports our conclusion. At the Shell Bar Section in Salt Lake Qarhan, Qaidam Basin [113] they are preserved in a silty clay (Fig 7) in which they occur in the form of accumulations of hundreds of complete, well preserved specimens.

### Human modifications

#### Grinding

**Fig 8:**
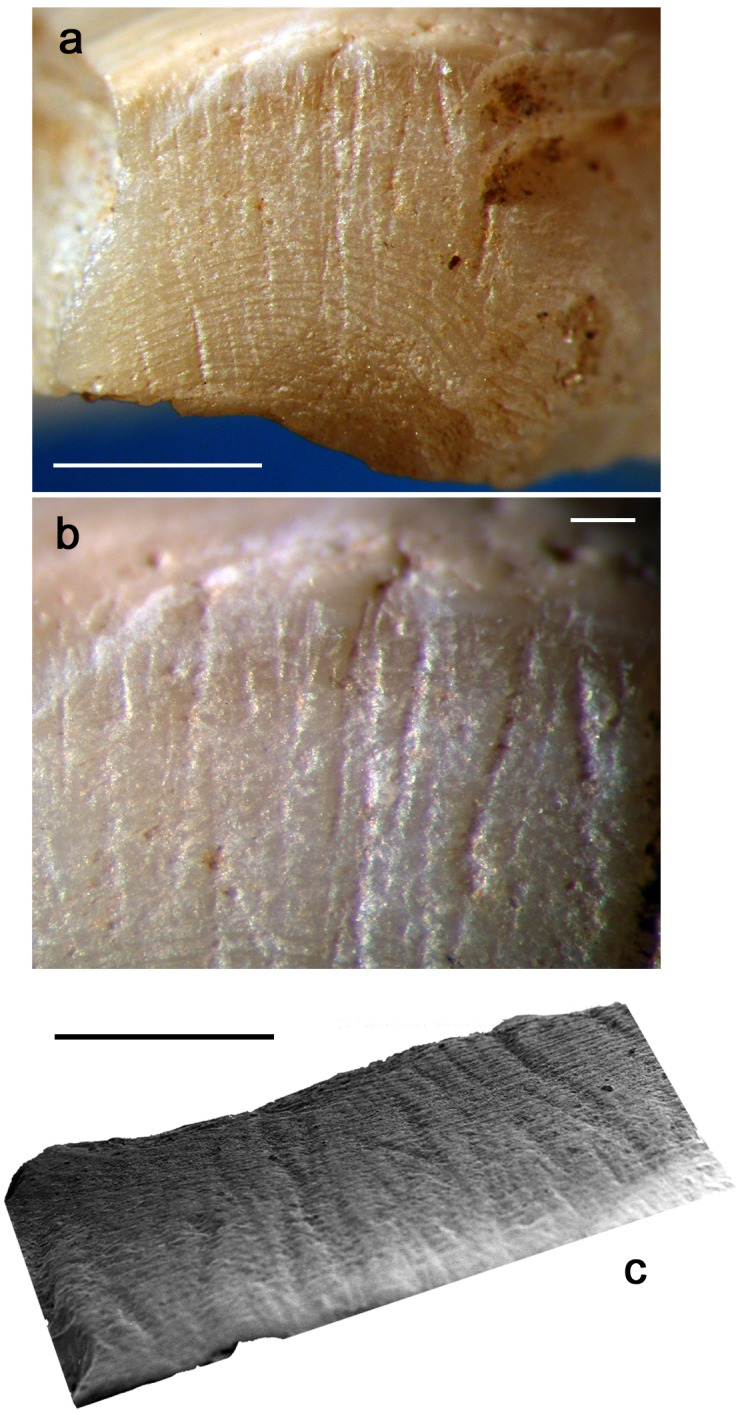
Anthropogenic modifications. **a.** facet produced by grinding, present on the umbo of the Shuidonggou shell; **b.** close-up view of the ground area; **c.** SEM photo of the striations. (a, c: scale = 1 mm; b: scale = 100 μm).

Application of this technique to the umbo of the archaeological specimen has created a flat facet covered by typical subparallel striations (Fig 8).

**Fig 9:**
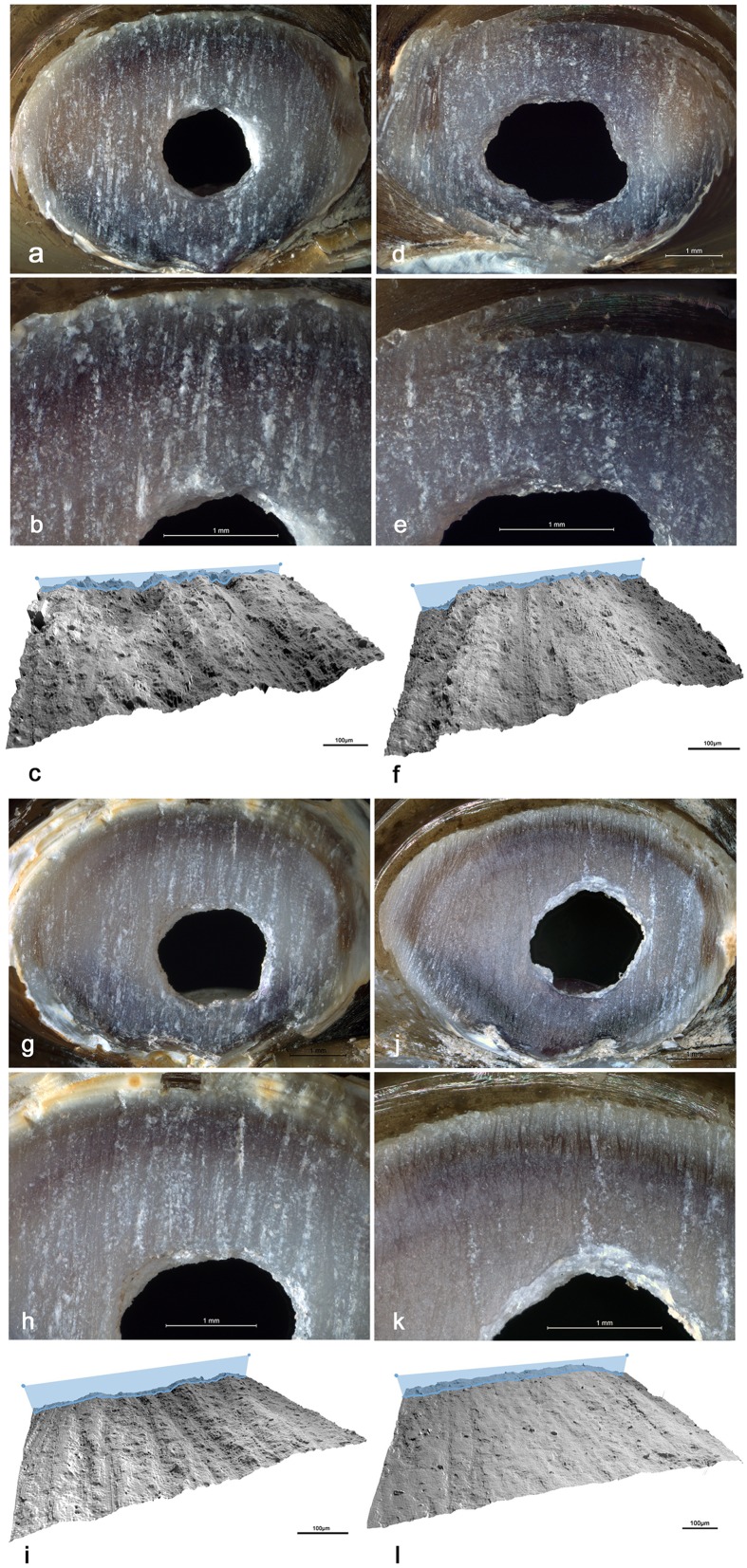
Experimental grinding. Microscopic images and 3D reconstructions obtained with a confocal microscope of facets produced by experimentally grinding *Corbicula fluminea* umbos on different grindstones. **a-c**. granite; **d-f**. basalt; **g-i.** coarse-grained sandstone; **j-l.** fine-grained sandstone.

The inclination of the facet indicates that the shell axis of symmetry was kept perfectly perpendicular to the grindstone during the work. The orientation of the striations on the facet and their morphology reveal, in turn, that the grinding was produced by a to-and-fro motion, and that the shell commissural plane, i.e. the plane separating the two valves, was kept perpendicular to the direction following which the shell was repeatedly displaced. The location and orientation of the facet relative to the shell morphology suggests that the area displaying the traces of grinding is the remnant of an originally much larger facet. The facet was produced with the aim of removing most of the umbo and opening into it a hole, a technique known at other Palaeolithic sites from China [88–89,74] and from all over the world [114–117] to transform bivalves into personal ornaments. Experimental application of the inferred technique and motion to well-preserved *Corbicula fluminea* umbos took 2–4 min to produce facets comparable in size and orientation to that visible on the Shuidonggou specimen (Fig 9). As expected, this technique also produced in the middle of the facet 2–3 mm wide sub-circular holes suitable for suspending the valve and using it as a personal ornament.

At microscopic scale, marked differences in appearance and roughness are observed between facets produced with grindstones made of different rocks (Fig 9). Rubbing *Corbicula* umbos on granite lower grindstone produces very irregular surfaces with no detectable striations (Fig 9a–9c). Exerted on a grindstone made of basalt, the same action produces a less rough surface crossed by shallow indistinct striations (Fig 9d–9f). The use of coarse-grained sandstone creates a more homogenous surface crossed by deep striations with irregular edges (Fig 9g–9i). Grindstones made of fine-grained sandstone produces instead even surfaces covered by distinct individual shallow striations (Fig 9j–9l). Such differences are due to the composition and hardness of the rock in which the grindstones are made. Granite and, to a lesser extent, basalt remove the shell calcitic layers by a mechanical action produced by contact with their surface asperities while sandstones create a wear resulting from both a plastic deformation at the contact between the two sliding surfaces and a mechanical abrasion produced by quartz and feldspar grains released by the rock matrix during the grinding process. The wear caused by the latter rock produces relatively flat surfaces associated with distinct striations whose dimensions reflect the size of the quartz/feldspar grains composing the sandstone. The wear pattern on the facet removing the umbo of the Shuidonggou valve (Fig 8) displays the association of features typical of a grinding produced on sandstone and more closely resembles that produced by experimentally grinding *Corbicula* umbos on a coarse-grained sandstone. This conclusion is consistent with available information on Shuidonggou Locality 2 lithics. Sandstone artifacts and pebbles are common at the site and represent one of the more frequently used lithic raw materials in layer CL3.

#### Engraving

**Fig 10:**
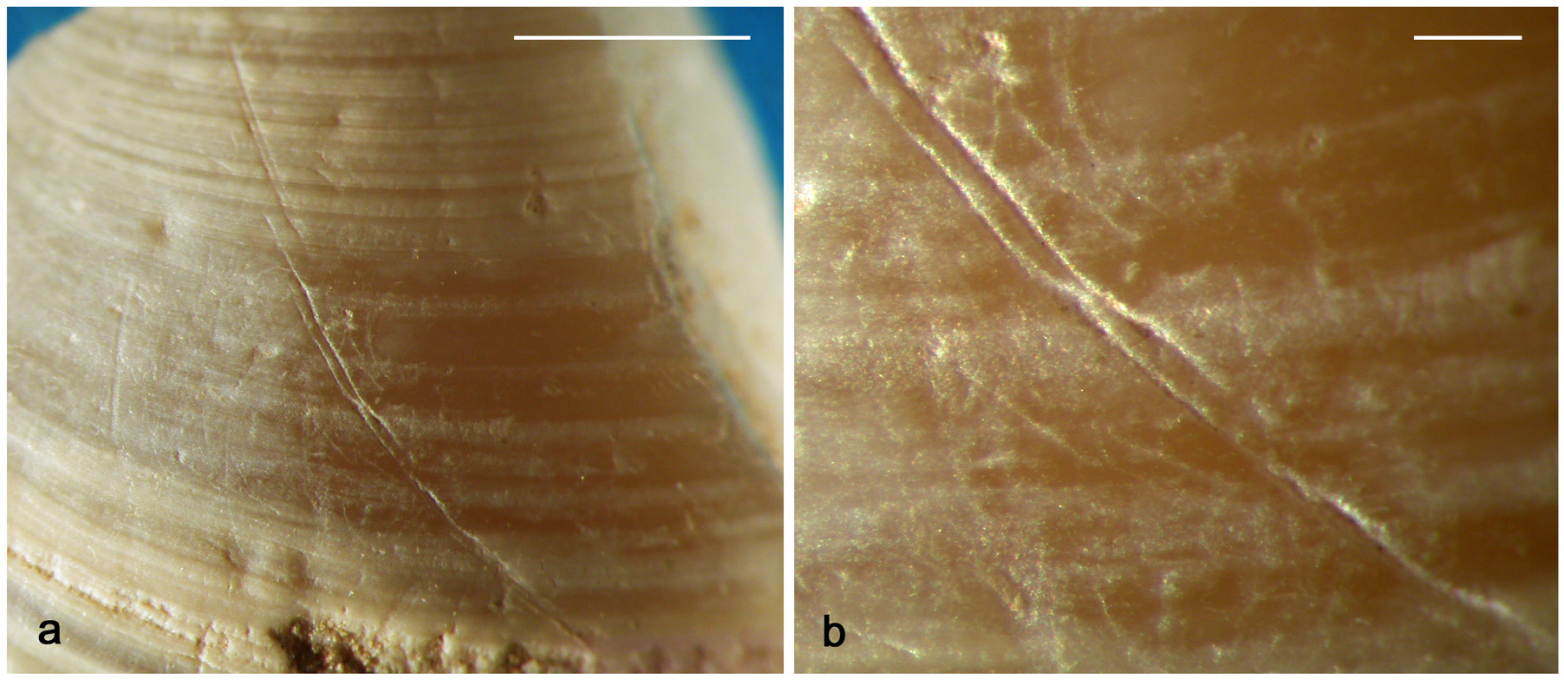
Engraving. **a.** incision on the exterior surface of the Shuidonggou shell; **b.** close-up view of the center of the incision (a: scale = 1 mm; b: scale = 100 μm).

**Fig 11:**
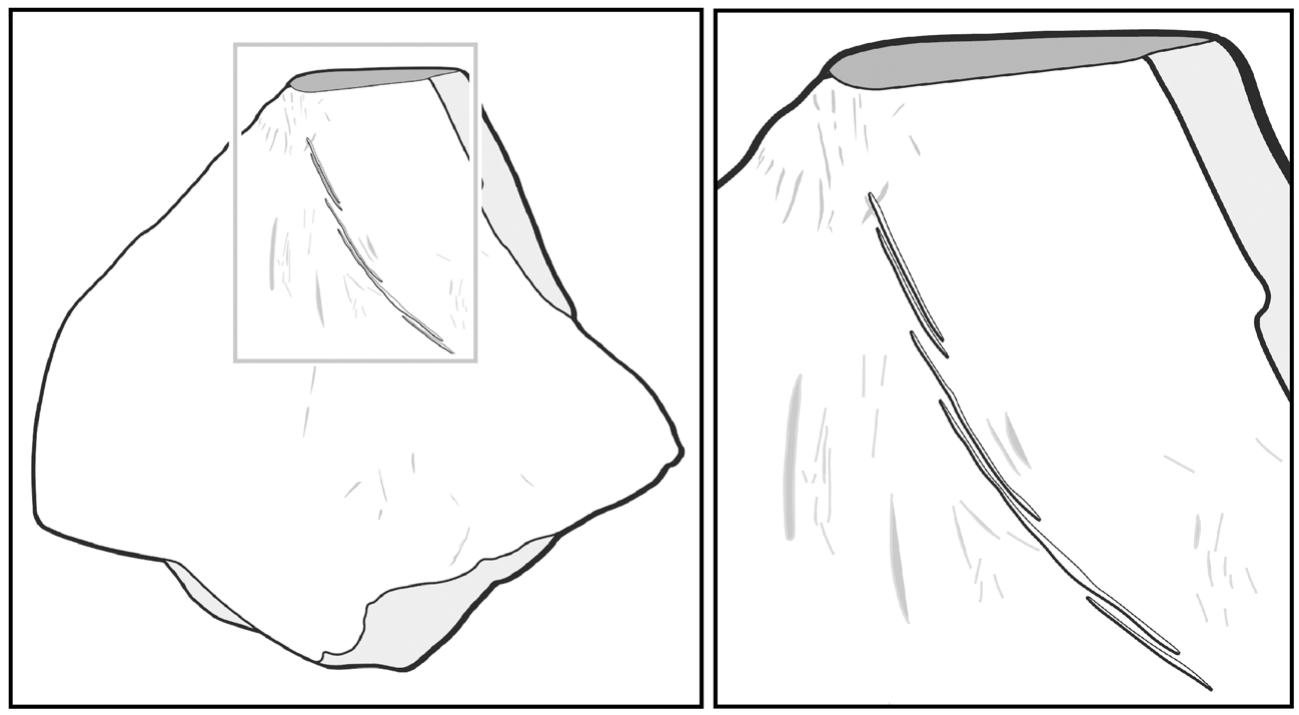
Tracing. Tracings of the shell bead with incised line (in black) and possible traces of utilisation (in gray).

The exterior surface of the valve is obliquely crossed by a curved line composed of five parallel, closely juxtaposed, striations (Figs 10 and 11).

Microscopic analysis of experimental engravings produced with a variety of stone tool types has demonstrated that this pattern results from the single passage of a point with a complex morphology that gradually changes the protuberances of the tip in contact with the engraved surface [118–119].

**Fig 12:**
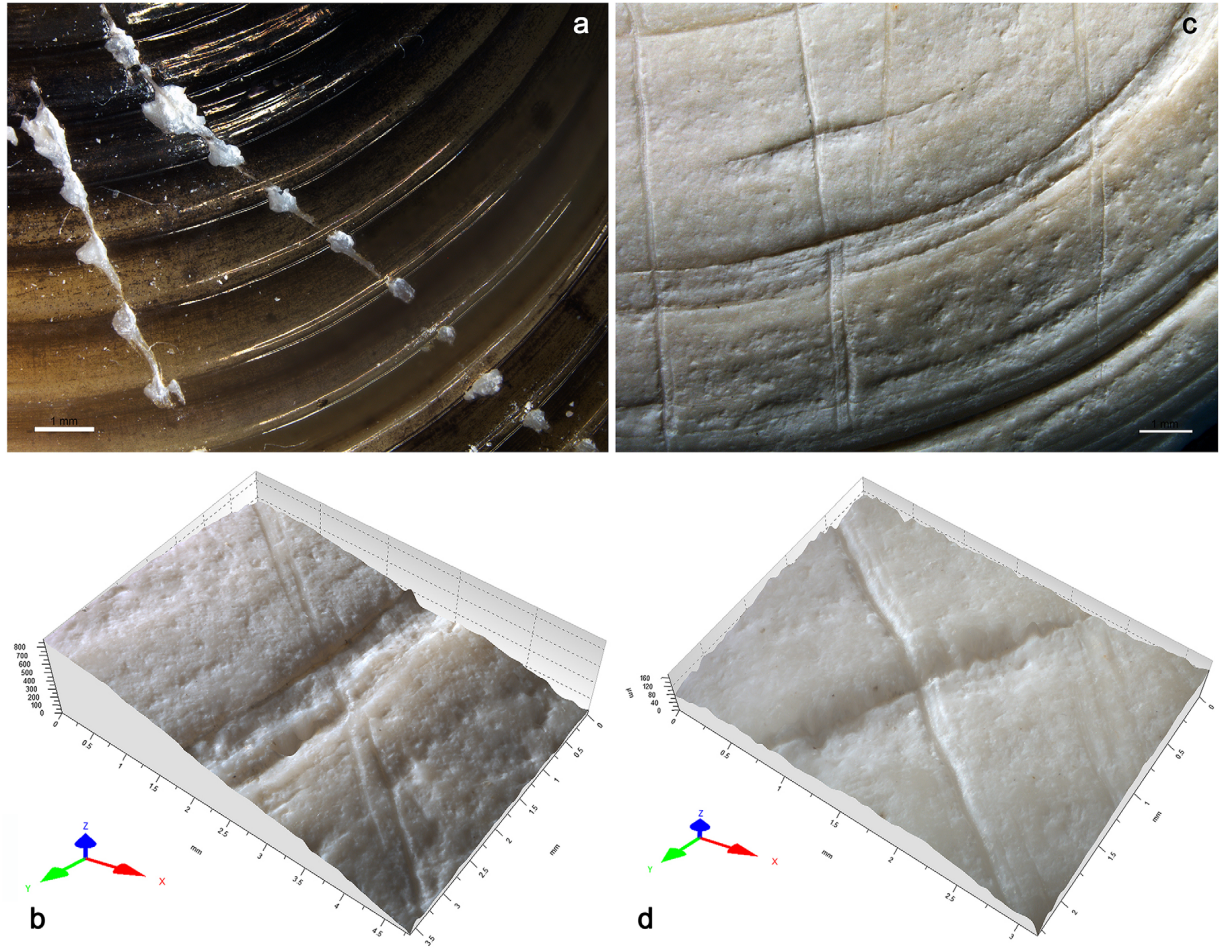
Experimental engraving. **a**. straight lines produced by experimentally engraving a *Corbicula* shell with pronounced growth ridges; **b-d**. momentary interruptions (b-c) and change of direction (d) of grooves produced by engraving lines on shells with less pronounced growth ridges (a, b: scale = 1mm).

Experiments conducted in the framework of this study reveal that engraving with a lithic point a valve bearing protruding growth ridges, like those typical of well-preserved *Corbicula* shells, produces discontinuous lines (Fig 12a).

This is due to the differential pressure exerted by the tool tip when crossing the wavy shell surface and the morphology of the tool tip that may not be sharp enough to effectively incise the bottom of concave areas. Engraving bivalves with less pronounced growth ridges result in continuous, often composite, lines that display either punctual interruptions (Fig 12b and 12c) or slight changes in direction (Fig 12d) when crossing concave areas. The latter feature, which repeatedly occurs on the line identified on the Shuidonggou shell (Fig 10), is more frequent when the tool is displaced slowly and without changing the pressure exerted on the shell surface.

#### Possible use-wear

No obvious use-wear due to threading is observed on the ground facet (Fig 8). This is to be expected since, as observed experimentally [120], a thread would preferentially wear, on a shell of this morphology, the bridge corresponding to the shell hinge, missing in our specimen.

**Fig 13:**
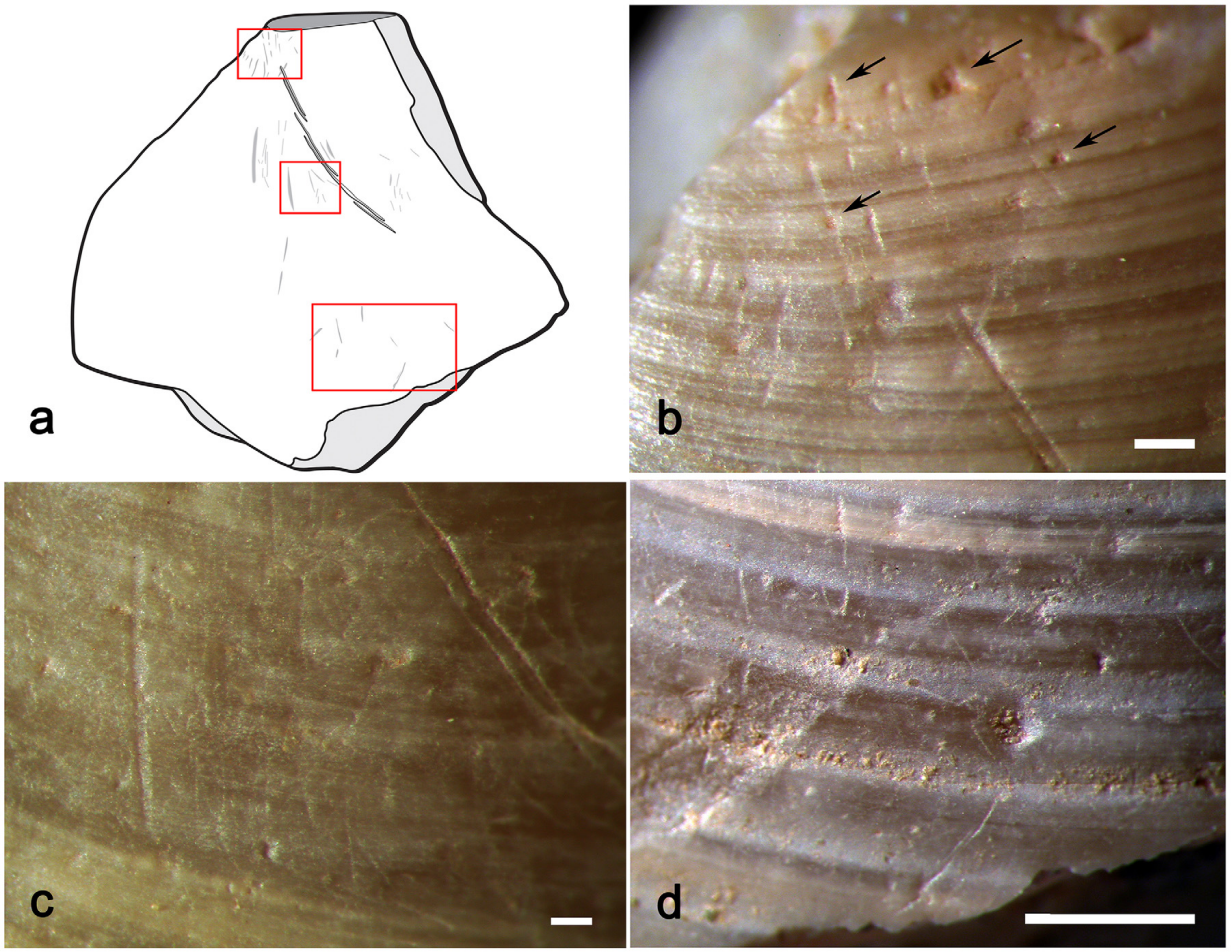
Microscopic analysis. a. Shell drawing with location of photos b-d; **b.** microstriations located between the ground facet and the engraved line; **c.** microstriations close to the engraved line with arrows indicating possible pigment residues; **d.** microstriations close to the valve distal fracture (b-d: scale = 100 μm).

The area located between the ground facet and the engraved line displays polishing and pitting associated with superficial shallow microstriations (Figs 11 and 13) Comparable microstriations, parallel to the valve main axis, are also recorded on both sides of the engraving and close to the valve distal fracture (Fig 13). The locations of these modifications are indicative of punctual repeated friction, post-depositional [121–122] or anthropogenic in origin. In the latter case the modifications may have been caused by contact with other objects during transport in a bag, from contact with clothes or personal ornaments integrated in the same beadwork.

#### Pigment residues

Microscopic residues of reddish material are detected at the bottom of the engraved lines and the pitting (Fig 13b). These residues are highly reflective and differ in color and grain size from the yellow sand in which the object was found. A firm identification of these residues as pigment originally covering the shell would require analysis of the residue and sediment surrounding the object that is not among the aims of the present study.

## Discussion

Information on the taphonomy of *C*. *fluminea* in different deposition environments suggests that the valve discovered at Shuidonggou Locality 2 was almost certainly collected at one of the numerous fossil or sub-fossil outcrops where well preserved valves of this species were easily available at the time of occupation of level CL3. Traces of grinding recorded on the umbo of the archaeological specimen, their experimental replication on modern valves of the same species leading to the opening of a hole, the use of the same technique at some other Palaeolithic sites from China and in other cultural and chronological contexts [114–117] to transform bivalves into personal ornaments, and the fact that no other uses apart from bead production are attested for this shell modification leave no doubt on the purpose of this action. The umbo was ground to open a hole in it, attach in all likeliwood a thread, and use the valve as a personal ornament. The location and extent of the ground surface on the archaeological specimen makes clear that the hole was already open and had a size comparable to or larger than that produced during our experiments, when the shell was lost or disposed. This indicates that the object is more likely a personal ornament whose umbo broke during use as a consequence of tension exerted by the twine than a preform accidentally damaged during the grinding process, and disposed before being used as a bead. This hypothesis is corroborated by the fact that no breakage of the umbo was produced during our grinding experiments, including those in which the shell was rubbed against a grindstone coarser than the one used to flatten the umbo of the Shuidonggou shell. Interpreting the line incised on the exterior of the shell is more problematic. Although, technically speaking, this is an engraved line, it does not display features (orientation, depth) providing compelling evidence of its deliberate nature and, if not highlighted by smearing pigment on it, this marking would have been virtually invisible to a person observing the bead when worn by somebody else. The line may have been accidentally produced when extracting the shell from a sub fossil outcrop or deliberately engraved. In the latter case it would more likely comply with an interpretation as a mark of ownership or a sign somehow related to a private or restricted symbolic use [123–126] rather than that of a design conceived for public display.

The identification of this object as a broken shell bead becomes particularly relevant when discussed in the light of available evidence on the site stratigraphy, dating, bead occurrence throughout the sequence, and current knowledge on the earliest evidence of bead use in Asia.

The vertical distribution of archaeological finds recorded at Locality 2 identifies CL2, where numerous OESB were recovered, and CL3, where the *C*. *fluminea* was found, as clearly distinct sub-horizontal concentrations of artifacts, separated by 40 to 50 cm of archaeologically sterile grayish yellow silt interpreted, as the reminder of the layers, as a combination of marginal bank and lake deposits [92, 127]. This indicates that the episode or episodes of occupation during which the broken shell became incorporated into the sediment clearly predate those in the course of which OESB were lost at the site. This implies either that human group accumulating CL2 exclusively wore OESB, and the group accumulating CL3 only wore shell beads, or that just one group, or both, wore the two ornament types but did not lose or dispose any while visiting Shuidonggou Locality 2. Ongoing research (Wei et al. in prep.) shows that the 83 OESB recovered thus far from CL2 include groups of beads made by different people, originally incorporated in distinct beadworks, lost at different times. This strongly suggests that a link exists between the number of OESB found at the site and the intensity of occupation, likely reflected by the number of discarded lithic artifacts. Although CL3 has yielded less than half (n = 873) of the lithics recovered from CL2 (n = 2114), their number is high enough, in the light of what we know about OESB deposition mode during the accumulation of CL2, to support the argument that at least some OESB should have been found in this concentration if the people accumulating CL3 were wearing them. The opposite, likely presence of shell beads in CL2 should have the visitors producing this concentration wore them, may be less true. The unicity of this find in CL3 may be due to the fact that only few of these objects were worn by CL3 visitors or that their loss was a rare occurrence. Since dozen if not hundred OESB are necessary to assemble a beadwork made of these tiny objects while a single or a few shells may suffice to create an effective beadwork, the probability of finding shell beads in the archaeological record may be much lower than that of finding OESB. In other words, shells may have been used as beads by the people who accumulated CL2 even if none of these objects was recovered during the excavation of this concentration, while it is less probable that OESB were used by the people who accumulated CL3. This supports the idea that significant differences may have existed in the type of beadwork used by the people responsible for the two accumulations. Such differences may have reflected distinct cultural affiliations or a cultural change within the same cultural tradition. In this respect, the time span separating the two occupations should not be underestimated. Inconsistencies and inversions in the ^14^C ages obtained for Locality 2 make it difficult to precisely establish when the shell bead was incorporated into the sequence. This probably happened before 34.6–34 cal kyr BP or 34.1–33.2 cal kyr BP, the two oldest and consistent ages obtained for overlying CL2 (Table 2), but it is problematic to say how long before considering the more recent ages (32.7–30.9 cal kyr BP) obtained for CL3.

An age of at least 33–34 cal kyr BP makes the *C*. *fluminea* recovered from CL3 one of the earliest instances of personal ornamentation, and the earliest example of a shell bead from China. The perforated teeth and disk from Xiaogushan layer 2 and 3, some of which may be as old as 43–46 cal kyr BP, are the only possibly earlier examples of this behavior in China. However, they come from a cave site deposit with higher chances of post-depositional disturbance, in which identified archaeological layers record very wide ^14^C age ranges, and little control exist, as argued by Qu et al. [84], on the relationship between dated samples and beads provenance. If confirmed by direct dating or discovery of similar objects in secure contexts, the production of perforated teeth at such an early period would indicate that the OESB and shell bead found at Shuidonggou Locality 2 represent a more recent bead tradition, which emerged after a phase in which personal ornaments similar to those found at sites from Siberia were used. The possibility of two asynchronous, chronologically overlapping bead traditions, one reflected by perforated teeth diffusing from Siberia, the other manifested by OESB coming from Mongolia, is a working hypothesis that needs to be tested in the future.

Uncertainty in the dating and site formation processes of many of these sites makes it difficult reaching firm conclusions. However, it is clear that once established, a bead tradition associating or alternating OESB and freshwater shells became a key feature of the Chinese Late Palaeolithic. This is not only indicated by the discovery of these beads types at sites of different periods (Shizitan, Hutouliang, Yujiagou), but also by the use, at these sites, of the same technique and motion—grinding of the umbo—to transform freshwater shell into beads.
